# Influence of Host Species, Location, and Aphid Prey on Microbial Diversity and Community Dynamics of Aphidophagous Ladybird Beetles in Guangxi, China

**DOI:** 10.1002/ece3.71036

**Published:** 2025-02-20

**Authors:** Mei‐Lan Chen, Yu‐Hao Huang, Li‐Qun Cai, Xiang‐Miao Qin, Xin‐Yi Meng, Hao‐Sen Li, Hong Pang

**Affiliations:** ^1^ School of Environmental and Life Sciences Nanning Normal University Nanning China; ^2^ Guangdong Key Laboratory of Animal Conservation and Resource Utilization, Guangdong Public Laboratory of Wild Animal Conservation and Utilization, Institute of Zoology Guangdong Academy of Sciences Guangzhou China; ^3^ State Key Laboratory of Biocontrol, School of Ecology Sun Yat‐sen University Shenzhen China

**Keywords:** 16S rRNA amplicons, aphid prey, aphidophagous ladybird beetles, microbial dynamics, symbiotic bacteria

## Abstract

Host species, locations, and diet can significantly impact microbial diversity and community in insects. Several ladybird beetles are known as key predators and potential biological control agents for aphids. However, there is limited understanding of how host species, locations, and aphid prey influence the microbial diversity and community of aphidophagous ladybird beetles in natural environments. In this study, we collected 74 samples of ladybirds and their aphid prey from various locations in Guangxi, China, and sequenced the 16S amplicons to investigate differences in their microbiomes. The dominant genera in the ladybird samples, *Bacteroides* and *Alistipes*, were rarely reported as predominant in other ladybird populations, indicating a unique genus‐level microbial community pattern in Guangxi. Alpha diversity indices and Bray–Curtis distances varied significantly among ladybird species. Abundance analysis revealed that the relative abundance of dominant bacteria in aphidophagous ladybirds differed significantly among different ladybird species and locations. Although the primary and facultative aphid symbionts differed among aphid samples from various populations and locations, they had minimal direct impact on the microbial community of the aphidophagous ladybirds, being sporadically detected in the corresponding predator samples. Our findings provide insights into the microbial communities of ladybirds and aphids in sympatric and distinct field environments, highlighting the plasticity of microbial abundance in aphidophagous ladybirds across different ladybird species and locations, as well as the low retention rate of specific aphid symbionts in ladybird predators.

## Graphical abstract

Analyses of the 16S rRNA amplicons of several ladybirds and their aphid prey from various locations in Guangxi, China reveal the plasticity of microbial abundance in aphidophagous ladybirds across different ladybird species and locations. Additionally, aphid symbionts are sporadically detected in the corresponding predator ladybirds, indicating their low retention rate in natural environments.

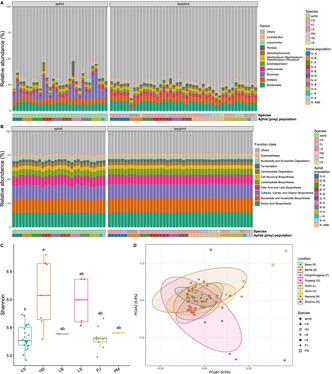

## 1 Introduction

The microbiome of various insects can be shaped by host taxa, diet, and local environment, as demonstrated in studies of termites (Bourguignon et al. 2018; Rahman et al. 2015; Sinotte et al. 2023; Marynowska et al. 2020), true bugs (Yang et al. 2023; Men et al. 2022), moths (Broderick et al. 2004; Jones et al. 2019), and beetles (Brunetti et al. 2022; Moldovan et al. 2023). In termites, gut microbiome members are typically inherited from the parent generation through trophallaxis or coprophagy and exhibit specialization among different taxa (Rahman et al. 2015; Bourguignon et al. 2018; Marynowska et al. 2020; Sinotte et al. 2023). Diet and local environment primarily affect the relative abundance of these microbiome members rather than their community composition (Sinotte et al. 2023; Marynowska et al. 2020; Rahman et al. 2015; Boucias et al. 2013). Additionally, an analysis of publicly available bacterial sequencing data found that insect species have a more significant impact on the structure and diversity of associated microbial communities than diet and sample origin, but weak phylogenetic signals support phylosymbiosis in only a few clades rather than across the entire insect phylogeny (Malacrinò 2022). These findings suggest that both the internal capacity of different insects to harbor microbes, such as their intracorporal physiology, and the external environments and their associated microbes can influence insect microbiomes. However, the internal capacity of insects to harbor microbes appears to have a greater impact, which needs to be further tested.

Ladybird beetles (Coleoptera: Coccinellidae) primarily feed on Sternorrhyncha (Hemiptera) pests, such as aphids and mealybugs (Hodek and Honěk 2009; Giorgi et al. 2009). Many ladybird species, particularly those in the Coccinellini tribe, are recognized as predators of aphids and are considered potential biological control agents (Nattier et al. 2021). Symbiotic microorganisms play crucial roles in ladybirds and other beetles, contributing to processes such as digestion, nutrition biosynthesis, antibiosis, cuticle formation, and interspecific competition (Huang et al. 2022; Li et al. 2024; Tang et al. 2024; Reis et al. 2020; Vogel et al. 2017; Anbutsu et al. 2017; Zhang et al. 2025). The composition of the microbiome in aphidophagous ladybirds can also influence the performance of these natural enemy insects (Costopoulos et al. 2014; Kovacs et al. 2017; Wang, Zhao, et al. 2024; Gao et al. 2023; Schmidtberg et al. 2019; Huang et al. 2022; Tang et al. 2024). Research has consistently shown that different diets have distinct impacts on the microbial communities of predatory ladybirds in laboratory settings, including an increased abundance of symbionts from corresponding prey (Du, Yang, et al. 2022; Huang et al. 2022, 2021; Wang, Gao, et al. 2024; Xie et al. 2024). Symbiotic bacteria from prey can persist in predatory ladybirds for extended periods or interact with predatory ladybirds (Du, Yang, et al. 2022; Du et al. 2023; Tang et al. 2024; Paula et al. 2015; Costopoulos et al. 2014; Kovacs et al. 2017; Pons et al. 2022; Wang, Zhao, et al. 2024; White et al. 2017). For example, *Serratia symbiotica* , a common facultative symbiont of aphids, can remain in the digestive tracts of ladybird predators after the complete digestion of aphid tissue, as confirmed by microscopy and DNA detection (Du, Yang, et al. 2022; Pons et al. 2022; Paula et al. 2015). It establishes a nearly neutral, co‐adaptive relationship with aphidophagous ladybirds, unlike its harmful effects on the survival and developmental performance of other ladybird species (Du, Yang, et al. 2022). This bacterium is subsequently excreted in feces, which enables it to infect aphids through contact with ladybirds (Du et al. 2023). Some other strains of *S. symbiotica* can also negatively affect predatory ladybirds by reducing their developmental and reproductive performance (Costopoulos et al. 2014; Kovacs et al. 2017; Wang, Zhao, et al. 2024). Additionally, facultative aphid symbionts like *Hamiltonella* and *Arsenophonus* have been reported to exhibit slower DNA decay than aphid DNA and, at some time nodes, to increase in abundance after aphid DNA degradation in predatory ladybirds (Paula et al. 2015). The retention of the aphid symbionts in ladybirds after digestion of host tissue suggests the potential for horizontal transfer between trophic levels, which still requires further verification through microscopic or RNA detection in ladybird tissue or cells. This retention phenomenon may contribute to the introduction of new members into the ladybird microbiomes, with unknown frequency and abundance.

In addition, host species and local environments influence the microbial communities of predatory ladybirds. The microbial diversity of two wild‐caught ladybird species, *Harmonia axyridis* and *Propylea japonica*, is higher compared to other common predatory insects in the same location, and their dominant bacteria are similar; however, the relative abundance of specific bacteria differs (Hu et al. 2022). Within the same species, *H. axyridis* from China and the USA exhibit significantly different microbial diversity, community composition, and abundance (Li et al. 2022). The influence of location may result from the limited migration capacity of ladybirds, as well as the effects of the surrounding environment, prey, and their microbiomes. Thus, in field environments, the microbial dynamics of aphidophagous ladybirds can be influenced by host species, geographic location, aphid prey, or a combination of these factors. However, the specific impacts and relative contributions of these factors to the microbial diversity and community of aphidophagous ladybirds in field environments remain largely unknown.

In order to obtain the overall landscapes of microbial community variation among the ladybirds of different species, locations, and aphid prey, we collected six aphidophagous ladybird species and their aphid prey from various locations in Guangxi, China. We sequenced the 16S rRNA amplicons of the bacterial communities in these samples to compare microbial diversity and composition among different host species, populations, and locations. These analyses aimed to explore the patterns of the microbiomes of ladybirds and aphids related to host species, populations, and geographic locations. Additionally, we examined how the microbial community of aphids affects the microbiomes of aphidophagous ladybirds.

## 2 Materials and Methods

Adult ladybirds and aphids were collected from eight locations in Guangxi, China (Tables 1 and S1). To ensure adequate DNA yield, three ladybird individuals or 15–30 aphid individuals were combined into a single sequencing sample. Total genomic DNA was extracted using the TIANamp Genomic DNA Kit (Tiangen Biotech, Beijing, China) following the manufacturer's protocol. DNA quality and quantity were assessed using a Nanodrop 1000 spectrophotometer (Thermo Fisher Scientific, Wilmington, USA). Only DNA samples with a 260:280 ratio between 1.8 and 2.0 and a 260:230 ratio between 2.0 and 2.5 were retained for sequencing. The nearly 420 bp V3–V4 region of the 16S rRNA gene was amplified using primers 338F (ACTCCTACGGGAGGCAGCA) and 806R (GGACTACHVGGGTWTCTAAT). The purified and quantified PCR products were sequenced on the Illumina HiSeq 2500 platform (Illumina, CA, USA), generating 250 bp paired‐end reads. High‐quality sequence reads were imported into QIIME2 v2023.5.1 (Bolyen et al. 2019), and denoising was performed using DADA2 v1.26.0 (Callahan et al. 2016) to obtain Amplicon Sequence Variants (ASVs). The taxonomy of ASVs was identified using the Naive Bayes classifier and reference datasets from the SILVA database (Quast et al. 2013) within QIIME2.

**TABLE 1:** List of aphid and ladybird samples collected from various locations in Guangxi, China. Sample codes represent abbreviations of the format “location‐host species‐prey population” for ladybird samples and “location‐host population A/B” for aphid samples. Detailed information on the sampling locations is listed in Table S1.

Sample code	Location	Host	Prey	Num
G‐CS‐A	Guigang	*Cheilomenes sexmaculata*	G‐A	3
G‐PJ‐A	Guigang	*Propylea japonica*	G‐A	3
G‐LS‐B	Guigang	*Lemnia saucia*	G‐B	3
G‐PM‐B	Guigang	*Platynaspis maculosa*	G‐B	1
G‐A	Guigang	*Aphis fabae*	—	3
G‐B	Guigang	*Aphis fabae*	—	3
F‐CS‐A	Fangchenggang	*Cheilomenes sexmaculata*	F‐A	3
F‐A	Fangchenggang	*Rhopalosiphum maidis*	—	3
B‐CS‐A	Beihai	*Cheilomenes sexmaculata*	B‐A	3
B‐PJ‐A	Beihai	*Propylea japonica*	B‐A	3
B‐CS‐B	Beihai	*Cheilomenes sexmaculata*	B‐B	3
B‐A	Beihai	Unknown aphid species	—	3
B‐B	Beihai	Unknown aphid species	—	3
Q‐CS‐A	Qinzhou	*Cheilomenes sexmaculata*	Q‐A	3
Q‐A	Qinzhou	Unknown aphid species	—	3
N‐CS‐AB	Nanning	*Cheilomenes sexmaculata*	N‐A, N‐B	3
N‐PJ‐AB	Nanning	*Propylea japonica*	N‐A, N‐B	3
N‐HD‐AB	Nanning	*Harmonia dimidiata*	N‐A, N‐B	3
N‐LB‐AB	Nanning	*Lemnia biplagiata*	N‐A, N‐B	1
N‐A	Nanning	*Rhopalosiphum maidis*	—	3
N‐B	Nanning	*Aphis gossypii*	—	3
H‐CS‐A	Hechi	*Cheilomenes sexmaculata*	H‐A	3
H‐HD‐A	Hechi	*Harmonia dimidiata*	H‐A	3
H‐LS‐A	Hechi	*Lemnia saucia*	H‐A	1
H‐A	Hechi	Unknown aphid species	—	3
L‐CS‐A	Guilin	*Cheilomenes sexmaculata*	L‐A	1
L‐A	Guilin	Unknown aphid species	—	1
S‐CS‐A	Baise	*Cheilomenes sexmaculata*	S‐A	1
S‐HD‐A	Baise	*Harmonia dimidiata*	S‐A	1
S‐A	Baise	Unknown aphid species	—	1

Downstream analyses based on the feature table were conducted using the R package microeco v1.8.0 (Liu et al. 2021). Specifically, ASVs classified as “mitochondria” or “chloroplast” were excluded, and the feature table was rarefied to 17,122 counts, corresponding to the minimum sample count. Alpha diversity of the microbial community was assessed using Shannon and Simpson indices. Alpha rarefaction curves were generated by randomly subsampling the ASV table with the R package mecodev v0.2.0 (Liu et al. 2021). Significance levels among different groups were tested using the Kruskal–Wallis rank sum test for all groups and Dunn's Kruskal–Wallis multiple comparisons for paired groups. *p*‐values were adjusted using Holm's step‐up procedure. Beta diversity was measured using Bray–Curtis distance metrics and visualized with principal coordinate analysis (PCoA). Differential tests of Bray–Curtis distances among the variables “species + location + aphid prey population” were performed in the ladybird samples using permutational multivariate analysis of variance (PERMANOVA) with 999 permutations via the R package vegan v2.6‐6.1 (Dixon 2003). For the aphid samples, because part of the aphid samples was not classified into species, samples were grouped into aphid populations, and the variables “location + population” were used in the PERMANOVA analysis. Significance levels of Bray–Curtis distances within the same ladybird species were assessed using the same methods as those for alpha diversity. Redundancy analysis (RDA) was employed to evaluate the relative contributions of “species + location + aphid prey population” and “location + population” to variations in bacterial community composition for ladybird and aphid samples, respectively. Additionally, envfit analysis was conducted to determine the contribution of the variables to the RDA model using the vegan package. Function prediction was performed using PICRUSt2 v2.5.3 (Douglas et al. 2020), and the functional abundances of MetaCyc pathway and functiones class were visualized using the microeco package.

The linear discriminant analysis (LDA) effect size (LEfSe) method (Segata et al. 2011) was utilized to identify taxa with significantly different abundances between aphid and ladybird samples. Taxa were deemed significant if they had a *p*‐value < 0.05 and an LDA score > 2.0. To identify taxa with significant abundance differences among groups due to varying locations and species, the Kruskal–Wallis rank sum test was performed after excluding taxa with relative abundances < 0.01%. The abundance of these taxa was then visualized.

In addition to the above analyses conducted using the microeco package, the R package FEAST v0.1.0 (Shenhav et al. 2019) was utilized for microbial source tracking to estimate the contribution of aphid samples to the origins of microbial communities in ladybird samples. Default parameter settings were used, with ladybird samples designated as sinks and the samples from their corresponding prey populations (Table 1) as sources.

## 3 Results

### 3.1 General Features and Characterization of Microbial Community and Functions

Using DADA2 (Callahan et al. 2016) in QIIME2 (Bolyen et al. 2019) for denoising and the microeco package (Liu et al. 2021) for contamination removal, we identified 17,122–54,114 features in aphid and ladybird samples collected from various locations in Guangxi, China (Tables 1 and S1). After rarefaction, a total of 51,571 ASVs were detected, of which 51,532 were taxonomically annotated. The rarefaction curve for each sample approached a plateau, indicating that the sequencing depth was adequate for accurately assessing the abundance of the bacterial community (Figure S1).

**FIGURE 1:**
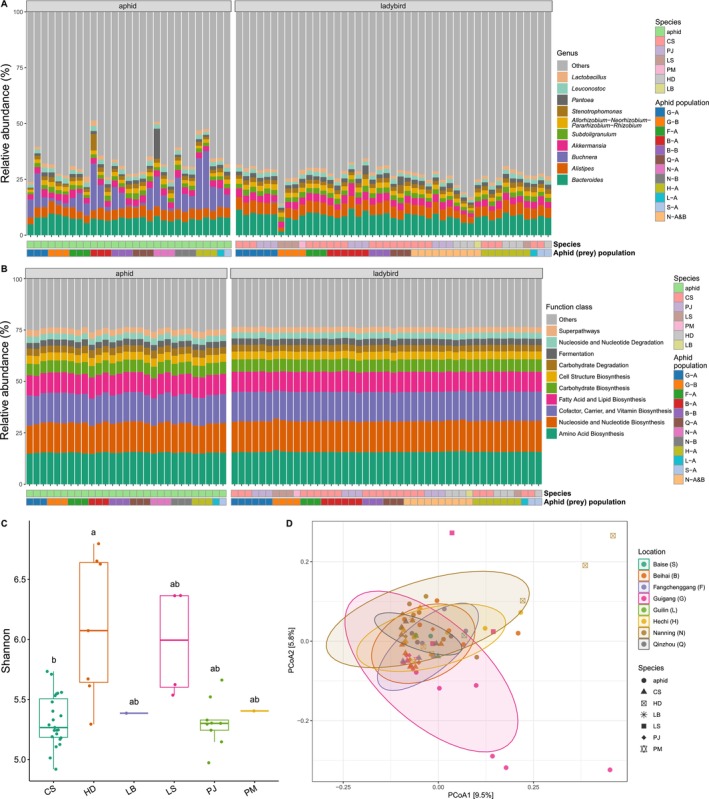
Microbial composition and diversity of aphid and ladybird samples collected from various locations in Guangxi, China. (A) Genus‐level taxonomic composition for each sample. (B) Function profiles for each sample. (C) Scattered boxplots of the Shannon index for different ladybird species. (D) Principal coordinate analysis (PCoA) based on Bray–Curtis distances for aphid and ladybird samples of different locations and species. Ellipses represent confidence intervals for different locations. Ladybird species name: CS, *Cheilomenes sexmaculata* ; HD, *Harmonia dimidiata* ; LB, *Lemnia biplagiata*; LS, *Lemnia saucia*; PJ, *Propylea japonica*; PM, *Platynaspis maculosa*. Aphid population codes represent abbreviations in the format “location‐host population A/B”. Detailed information on sample codes is provided in Table 1.

The top three most abundant phyla in both ladybird and aphid samples were the same, including Proteobacteria (23.82% and 32.35%, respectively), Bacteroidetes (20.78% and 18.28%, respectively), and Firmicutes (12.92% and 11.96%, respectively) (Figure S2). The most prevalent genera in ladybird samples were *Bacteroides* (8.43%), *Alistipes* (4.76%), and *Akkermansia* (3.16%), while *Buchnera* (8.10%), *Bacteroides* (7.34%), and *Alistipes* (4.46%) had the highest abundance in the aphid samples (Figure 1A). Apart from *Buchnera*, a primary symbiotic bacterium in aphids (Baumann 2005), other abundant genera were present in all aphid and ladybird samples. *Buchnera* was consistently found in aphid samples with a relatively high abundance (0.72%–25.60%), whereas it was detected in only a few ladybird samples at low abundance (< 0.54%).

Functional predictions generated using PICRUSt2 (Douglas et al. 2020) indicated that the pathways under “Amino Acid Biosynthesis” (aphid: 15.34%, ladybird: 15.72%), “Nucleoside and Nucleotide Biosynthesis” (aphid: 14.30%, ladybird: 14.97%), and “Cofactor, Carrier, and Vitamin Biosynthesis” (aphid: 14.00%, ladybird: 14.26%) were most abundant in both aphid and ladybird microbiomes (Figure 1B). The top three specific pathways in aphid microbiomes were “aerobic respiration I (cytochrome c)” (1.18%), “pyruvate fermentation to isobutanol (engineered)” (0.79%) and “gondoate biosynthesis (anaerobic)” (0.79%). These same pathways were also the most abundant in ladybird microbiomes, with slightly higher proportions of 1.31%, 0.84%, and 0.84%, respectively (Figure S3).

### 3.2 Microbial Diversity and Composition Across Different Locations, Species, and Prey

Bacterial alpha diversity for each sample was assessed using Shannon and Simpson indices. The significance of differences among various groups according to locations and species was evaluated. The results showed no significant differences in Shannon and Simpson indices among the groups (Figures S4 and S5). However, when comparing species alone, both Shannon and Simpson indices for *Harmonia dimidiata* were significantly higher than those for *Cheilomenes sexmaculata* (Figures 1C and S6).

Community dissimilarity was quantified using the Bray–Curtis distance and visualized through PCoA (Figure 1D). PERMANOVA analyses revealed significant differences in Bray–Curtis distances among different locations (33.48% of the variance explained, *p*‐value = 0.002) and aphid populations (12.05% of the variance explained, *p*‐value = 0.034) in the aphid samples (Table 2). For the ladybird samples, only weak significance (*p*‐value = 0.094) of the differences was detected among different ladybird species, which explained 14.84% of the variance (Table 2). Moreover, intraspecific Bray–Curtis distances within *H. dimidiata* and *Lemnia saucia* were significantly greater than those within *P. japonica* and *C. sexmaculata* (Figure S7).

**TABLE 2:** Effects of host species, location, population, and prey on the aphid and ladybird microbiomes, based on permutational multivariate analysis of variance (PERMANOVA) analyses using Bray–Curtis distances.

Sample	Factor	*R* ^2^	*F*	*p*
Aphid	Location	0.335	1.581	0.002
Aphid	Population	0.120	1.327	0.034
Ladybird	Host species	0.148	1.367	0.094
Ladybird	Location	0.162	1.063	0.238
Ladybird	Aphid prey population	0.039	0.887	0.620

Based on the adjusted *R* ^2^ from RDA analysis, 30.37% of the variation in aphid microbial community composition was explained by location and population variables, while 22.48% of the variation in ladybird microbial community composition was explained by ladybird species, location, and prey population variables (Figures S8 and S9). The contributions of location (*R* ^2^ = 0.58, *p*‐value = 0.005) and population (*R* ^2^ = 0.66, *p*‐value = 0.002) to the aphid microbiomes, as well as ladybird species (*R* ^2^ = 0.26, *p*‐value = 0.018) to the ladybird microbiomes, were significant according to the envfit analysis. In the RDA analysis of the aphid samples, *S. symbiotica* and *Regiella insecticola* were associated with the samples collected from Guigang, the secondary endosymbiont of *Bemisia tabaci* (belonging to *Hamiltonella*) was linked to one population of Beihai, and *Buchnera aphidicola* ( *Aphis gossypii* ) was associated with the population from Hechi (Figure S8). The RDA analysis of the ladybird samples revealed that *Bacteroides dorei* was associated with ladybirds feeding on a specific aphid population from Guigang (Figure S9).

### 3.3 Analysis of Bacterial Taxa With Significant Abundance Differences Among Host Species and Locations

**FIGURE 2:**
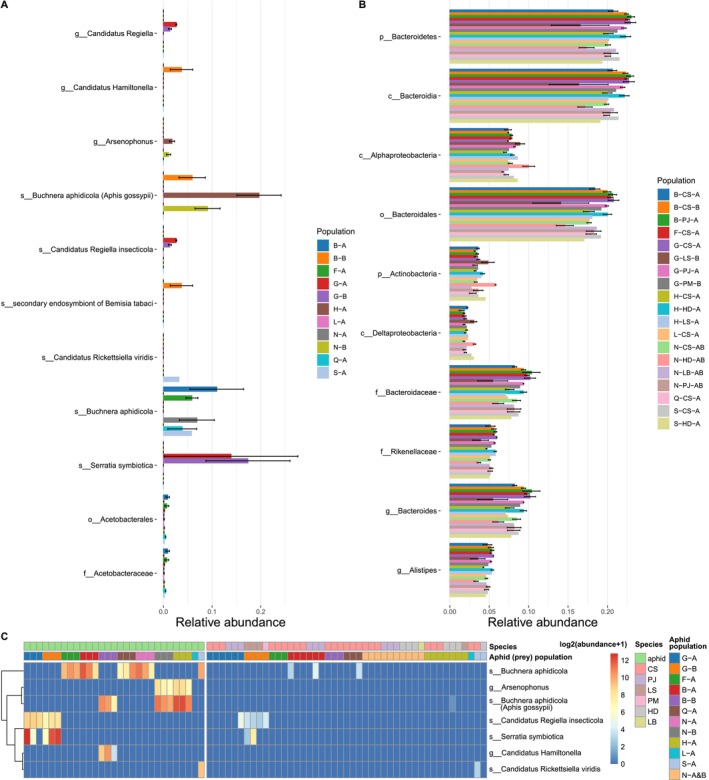
Taxa with significantly varying abundances across locations and species, based on the Kruskal–Wallis rank sum test. (A) Taxa with significant variations in abundance among locations and populations in the aphid samples. (B) Taxa with significant variations in abundance among locations, species and prey in the ladybird samples. Only the top 10 taxa with the highest abundances are shown. (C) Abundance of taxa with significant variations among the aphid samples in all aphid and ladybird samples. Population codes represent abbreviations in the format “location‐host species‐prey population” for ladybird samples and “location‐host population A/B” for aphid samples. Ladybird species name: CS, *Cheilomenes sexmaculata* ; HD, *Harmonia dimidiata* ; LB, *Lemnia biplagiata*; LS, *Lemnia saucia*; PJ, *Propylea japonica*; PM, *Platynaspis maculosa*. Location: B, Beihai; F, Fangchenggang; G, Guigang; H, Hechi; L, Guilin; N, Nanning; Q, Qinzhou; S, Baise. Detailed information on sample codes is provided in Table 1.

Two strains of *B. aphidicola* were significantly more abundant in aphid samples compared to ladybird samples, as determined by the LEfSe method (Segata et al. 2011) (Figure S10). At the genus level, *Bacteroides*, *Pantoea*, *Enterobacter*, *Pseudomonas*, *Achromobacter*, and *Kosakonia* were more prevalent in ladybird samples. The Kruskal–Wallis test revealed several taxa with significantly different abundances among locations and populations within aphid samples. These included two strains of *B. aphidicola* associated with different locations, *S. symbiotica* and *R. insecticola* prevalent in aphids collected from Guigang, *Hamiltonella* (secondary endosymbiont of *B. tabaci* ) in a population of Beihai, *Arsenophonus* in Hechi population and a population of Nanning, and *R. viridis* in Baise population (Figure 2A). In ladybird samples, the genera with the greatest variability in abundance were *Bacteroides* and *Alistipes* (Figure 2B). For the taxa with significantly different abundances among the aphid samples, two strains of *B. aphidicola* , *S. symbiotica* , *R. insecticola*, and *R. viridis* were identified in several ladybird species (i.e., *C. sexmaculata* , *P. japonica* , *H. dimidiata* , *L. saucia*, and *Platynaspis maculosa*) collected from the same locations as the aphid samples containing these symbionts (Figure 2C). However, only *S. symbiotica* was found in appreciable abundance in both aphid samples and a sample of *L. saucia* (relative abundance: 1.00%) collected from Guigang.

### 3.4 Microbial Source Tracking From Aphids to Ladybirds

**FIGURE 3:**
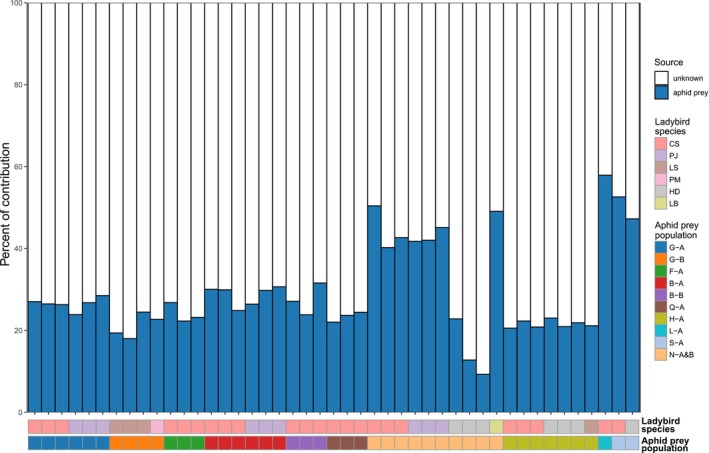
Putative contribution of microbial communities in aphid samples to surrounding ladybird samples, based on microbial source tracking using the FEAST package. Ladybird species name: CS, *Cheilomenes sexmaculata* ; HD, *Harmonia dimidiata* ; LB, *Lemnia biplagiata*; LS, *Lemnia saucia*; PJ, *Propylea japonica*; PM, *Platynaspis maculosa*. Aphid population codes represent abbreviations in the format “location‐host population A/B”. Detailed information on sample codes is provided in Table 1.

Based on the results of the FEAST package (Shenhav et al. 2019), bacteria from corresponding aphid prey contributed 9.30%–57.89% to the origins of the microbial communities of ladybird samples (Figure 3).

## 4 Discussion

In this study, we sequenced and compared the microbiomes of ladybird and aphid samples from different locations. The dominant phyla identified were Proteobacteria, Bacteroidetes, and Firmicutes, which were consistent with findings from other studies on the microbial communities of *H. axyridis* and *P. japonica* (Zhao et al. 2020; Xie et al. 2024; Lu et al. 2024; Hu et al. 2022; Gao et al. 2021; Dudek et al. 2017; Du, Xue, et al. 2022). However, the genus *Bacteroides*, which was dominant in our ladybird samples, has only been reported as one of the dominant genera in wild‐caught *H. axyridis* and *P. japonica* from Henan, China (Hu et al. 2022). Additionally, another dominant genus *Alistipes* has rarely been reported as abundant in ladybirds. These observations suggest a locally specific genus‐level microbial pattern in ladybirds distributed in Guangxi, China. Previous studies have identified various genera as dominant bacteria in aphidophagous ladybirds (e.g., *H. axyridis* , *P. japonica* , *Coccinella septempunctata* ), including *Staphylococcus*, *Enterobacter*, *Glutamicibacter*, *Acinetobacter*, *Enterococcus*, *Serratia*, *Romboutsia*, *Escherichia*‐*Shigella*, *Terrisporobacter*, *Lactobacillus*, *Lactococcus*, *Sphingomonas*, *Ochrobactrum*, *Rhodococcus*, *Rhodanobacter*, and *Methylovirgula* (Zhao et al. 2020; Xie et al. 2024; Lu et al. 2024; Hu et al. 2022; Gao et al. 2021; Dudek et al. 2017; Du, Xue, et al. 2022; Du, Yang, et al. 2022). The diversity of these dominant genera across different ladybird populations highlights the high plasticity of microbiomes in aphidophagous ladybirds.

Regarding the predicted functions of the ladybird microbiomes, the most abundant functions were the biosynthesis of amino acids, nucleosides, nucleotides, cofactors, carriers, and vitamins, which are also important in their aphid prey (Oliver et al. 2010; Baumann 2005). Furthermore, studies on *H. axyridis* have found that these functions are part of the most abundant functions in the microbiomes of this species, and their abundance is associated with feeding on aphids (Xie et al. 2024; Sun et al. 2024; Wang, Gao, et al. 2024). If these predicted functions are valid for the ladybird microbiomes, they suggest that the biosynthesis of these nutrients probably plays a crucial role in the aphidophagous ladybirds. However, the transient bacteria or their residual DNA from undigested aphid tissue have no actual function in the ladybirds, though their functions are predicted. The true functions of ladybird microbiomes should be further investigated through experiments in the future.

Our study revealed significant differences in microbial diversity and community composition among ladybird and aphid populations from different locations and species, consistent with findings for other insects (Malacrinò 2022). Both alpha diversity (measured by Shannon and Simpson indices) and beta diversity (assessed by intraspecific Bray–Curtis distances) varied significantly across ladybird species. Specifically, the alpha diversity of *H. dimidiata* was significantly higher compared to *C. sexmaculata* , contrasting with the lack of difference between *H. axyridis* and *P. japonica* (Hu et al. 2022). Additionally, Bray–Curtis distances between samples within the same species of *H. dimidiata* and *L. saucia* were significantly higher than those of *P. japonica* and *C. sexmaculata* , indicating higher intraspecies dissimilarity. A similar pattern was observed in previous research, which noted lower dissimilarity for *P. japonica* compared to *H. axyridis* (Hu et al. 2022). Furthermore, ladybird species and location varied the abundance of dominant genera such as *Bacteroides* and *Alistipes*. These findings suggest that microbial variations in ladybirds are influenced by both physiological and geographical isolation, likely due to endosomatic and local environmental conditions. However, PERMANOVA and envfit analysis revealed that only ladybird species significantly contributed to the variance in their microbiomes, suggesting that endosomatic factors may have a more dominant role. Unlike insects with primary symbiotic bacteria, such as hemipterans, which show taxonomic influence on microbial members (Yang et al. 2023), our study indicates that these microbial variations are primarily related to abundance rather than microbial composition. This is possibly related to the absence of primary symbiotic bacteria or other closely associated bacteria in the ladybirds.

Different prey can significantly influence the microbial diversity and community structure of predatory ladybirds. The microbiomes of predatory ladybirds vary notably when they consume aphids, mealybugs, moth eggs, or psyllids (Du, Yang, et al. 2022; Huang et al. 2022, 2021; Wang, Gao, et al. 2024). The effects of different aphid prey, including aphid species themselves and populations with different symbiotic bacteria, on ladybird microbiomes remain largely unexplored. Unfortunately, the lack of precise species information hinders assessment of the direct impacts of aphid species in our study. Consequently, we focus on the effects of aphid symbiotic bacteria on ladybird microbiomes. In the aphid prey samples, the primary symbiotic bacterium *Buchnera* was widely detected and present in relatively high abundance, consistent with previous studies (Huang et al. 2022; Wang, Gao, et al. 2024; Oliver et al. 2010). The abundance of bacteria in the aphid samples varied significantly across different populations and locations. Notable symbionts included two strains of *B. aphidicola* , *S. symbiotica* , *R. insecticola*, *Hamiltonella*, *Arsenophonus*, and *R. viridis* . These bacteria, which are either primary or facultative symbionts, confer various benefits to aphids, such as defense against enemies and pathogens, resistance to heat stress, and enhanced nutrient utilization (Oliver et al. 2010; Baumann 2005). The symbiotic bacteria were present in all samples within the same populations with relatively high abundance, rather than showing sporadic distribution. The presence of two strains of *B. aphidicola* in distinct aphid populations, even within the same locations, suggests that these strains are likely to be exclusive to specific populations or species. In Guigang, both *S. symbiotica* and *R. insecticola* were observed in different populations. The presence of *S. symbiotica* in different populations supports that it has a strong capacity for transfer among aphids, possibly through direct contact between aphids, ladybirds, and the surrounding environment (Du et al. 2023; Pons et al. 2022). The variation in aphid symbiotic bacteria across different populations and locations allows us to investigate the impact of prey symbiotic bacteria on the microbiomes of aphidophagous ladybirds.

In the ladybird samples, microbial source tracking indicated that 9.30%–57.89% of the ladybird microbial community's bacteria originated from their aphid prey. However, the microbial composition did not differ significantly among ladybirds fed on different aphid populations, despite those aphid populations exhibiting significantly distinct microbiomes. Additionally, the primary or facultative symbiotic bacteria from aphid prey were not detected as bacteria with significantly different abundance in the ladybirds. Instead, these symbiotic bacteria, which varied in abundance in the aphid prey, appeared sporadically in some of the corresponding ladybird predator samples. This distribution could be due to transient flora introduced through diet or persistence in the ladybirds after aphid digestion. While *B. aphidicola* and *R. insecticola* from aphid prey are likely to decay rapidly after ingestion by ladybirds (Du, Yang, et al. 2022; Paula et al. 2015), these two aphid symbionts were found in low abundance in the ladybird samples. *B. aphidicola* , closely associated with the aphid hosts due to its intracellular location and reduced genome, is unlikely to survive in other hosts (Baumann 2005). Therefore, the low abundance of *B. aphidicola* detected in the ladybird samples is likely due to undigested aphid tissue ingested shortly before sample collection. *R. insecticola* may adversely affect the survival and performance of ladybirds (Kovacs et al. 2017), but its low abundance could also be attributed to undigested aphid tissue, and its persistence in ladybirds needs to be further tested. Although facultative aphid symbionts *Hamiltonella* and *Arsenophonus* have been shown to persist in ladybirds (Paula et al. 2015), and *Arsenophonus* may be obtained from scale insects to predatory ladybirds with relatively high abundance in a period of time (Tang et al. 2024), neither genus was detected in our ladybird samples. *S. symbiotica* is a facultative aphid symbiont that has been verified to have the ability to persist and play a relatively harmless role in aphidophagous ladybirds (Du, Yang, et al. 2022; Paula et al. 2015). In our study, *S. symbiotica* was also detected in our ladybird samples, with only one sample showing appreciable abundance, consistent with previous findings of low prevalence in wild‐caught ladybirds (Pons et al. 2022). This suggests the possibility that retention of *S. symbiotica* may occur in natural settings, albeit at low frequency. However, the presence of transient flora and their residual DNA cannot be ruled out by our data, indicating that retention rates may be even lower in actual field environments.

In conclusion, this study investigated the comprehensive influence of multiple factors on the microbiomes of aphidophagous ladybirds in sympatric and distinct field environments. The results revealed that both the microbial diversity and abundance in aphidophagous ladybirds are primarily influenced by the species of ladybird, with geographical locations also playing a role in microbial abundance. These findings support the high plasticity of aphidophagous ladybird microbiomes in relation to host species and locations, similar to other insects. In addition, we found that the distribution patterns of primary and facultative symbiotic bacteria from aphid prey varied among different populations and locations. However, these aphid symbionts had only a minor effect on the microbial community of aphidophagous ladybirds in the field, with sporadic detection and low abundance. The limitations of our study prevented a comprehensive explanation of the differences in microbial communities across host species and locations. To enhance the understanding of these influencing mechanisms, it is essential to record and analyze additional factors such as intracorporal pH, altitude, current temperature, humidity, and plant substrates alongside the microbial abundance data. Regarding the impact of aphid bacteria on the microbiomes of their ladybird predators, our data could not exclude the possibility of transient flora and their residual DNA. Therefore, we cannot support the long‐time persistence of aphid symbionts in ladybirds or the hypothesis of horizontal bacteria transfer between aphid prey and ladybirds based on our current data. Future research should employ microscopic or RNA detection techniques with starvation treatment to investigate this hypothesis. In addition to aphid prey, other potential sources such as surrounding insects, plants, and soil are worthy of consideration, as observed in other insects (Shan et al. 2024). Future studies should also analyze the microbiomes of these additional sources to assess their direct impact.

## Author Contributions

**Mei‐Lan Chen:** conceptualization (equal), data curation (equal), formal analysis (supporting), funding acquisition (equal), investigation (equal), methodology (supporting), project administration (equal), resources (lead), supervision (equal), validation (equal), visualization (supporting), writing – original draft (equal), writing – review and editing (equal). **Yu‐Hao Huang:** conceptualization (equal), data curation (equal), formal analysis (lead), investigation (supporting), methodology (equal), project administration (equal), software (lead), supervision (equal), validation (equal), visualization (lead), writing – original draft (equal), writing – review and editing (equal). **Li‐Qun Cai:** data curation (supporting), investigation (equal), methodology (equal), resources (supporting). **Xiang‐Miao Qin:** data curation (supporting), investigation (equal), resources (equal). **Xin‐Yi Meng:** data curation (supporting), investigation (equal), resources (equal). **Hao‐Sen Li:** conceptualization (equal), data curation (equal), formal analysis (supporting), funding acquisition (equal), methodology (equal), project administration (equal), resources (supporting), supervision (equal), validation (equal), visualization (supporting), writing – original draft (equal), writing – review and editing (equal). **Hong Pang:** conceptualization (supporting), funding acquisition (equal), project administration (supporting), validation (equal), writing – review and editing (equal).

## Conflicts of Interest

The authors declare no conflicts of interest.

## Supporting information


Appendix S1

## Data Availability

Raw reads of the 16S rRNA amplicons were deposited in the NCBI SRA database (BioProject accession: PRJNA763096).
