# Supplementary material for: Influence of Host Species, Location, and Aphid Prey on Microbial Diversity and Community Dynamics of Aphidophagous Ladybird Beetles in Guangxi, China
Source: Ecol Evol. 2025 Feb 20;15(2):e71036. doi: 10.1002/ece3.71036 (PMC11842868; doi:10.1002/ece3.71036)
Supplement: Supplementary file 1 — Appendix S1 [file ECE3-15-e71036-s001.docx]

Table S1 Detailed information on the sampling locations of ladybirds and aphids collected from the wild in Guangxi, China.

| Location ID | Sampling location | Longitude | Latitude |
| --- | --- | --- | --- |
| G | Pingnan County, Guigang City, Guangxi | 110° 39’ 38.660” E | 23° 17’ 6.025” N |
| F | Fangcheng District, Fangchenggang City, Guangxi | 108° 21’ 33.070” E | 21° 46’ 38.111” N |
| B | Hepu County, Beihai City, Guangxi | 109° 13’ 4.361” E | 21° 38’ 9.953” N |
| Q | Qinnan District, Qinzhou City, Guangxi | 108° 41’ 6.850” E | 21° 59’ 10.828” N |
| N | Wuming District, Nanning City, Guangxi | 108° 17’ 22.801” E | 23° 10’ 55.531” N |
| H | Donglan County, Hechi City, Guangxi | 107° 24’ 1.854” E | 24° 19’ 56.870” N |
| L | Gongcheng Yao Autonomous County, Guilin City, Guangxi | 110° 54’ 45.385” E | 24° 42’ 48.784” N |
| S | Pingguo City, Baise City, Guangxi | 107° 33’ 8.208” E | 23° 17’ 37.954” N |


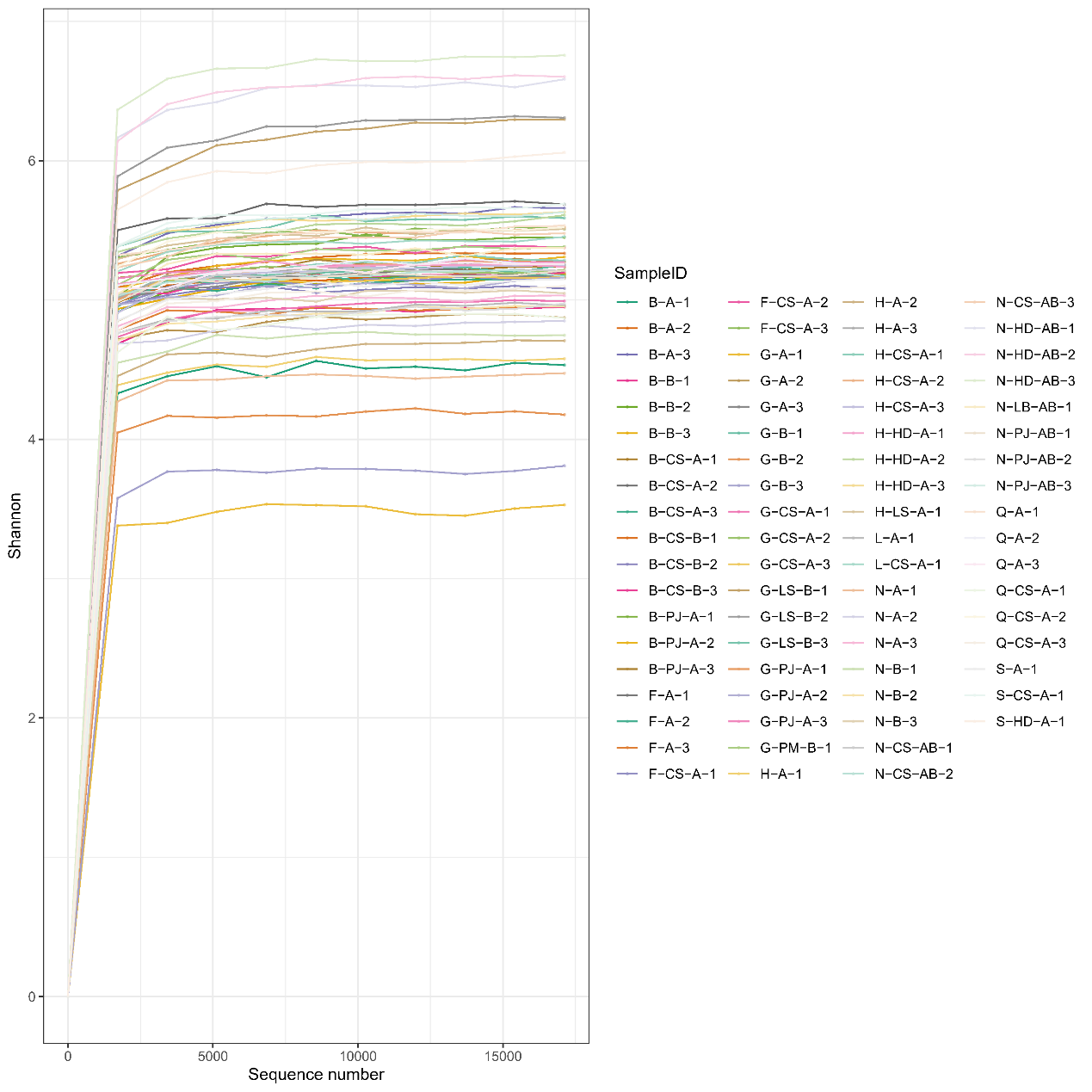


Figure S1 Rarefaction curves for aphid and ladybird samples collected from various locations in Guangxi, China. Detailed information on sample codes is provided in Table 1.


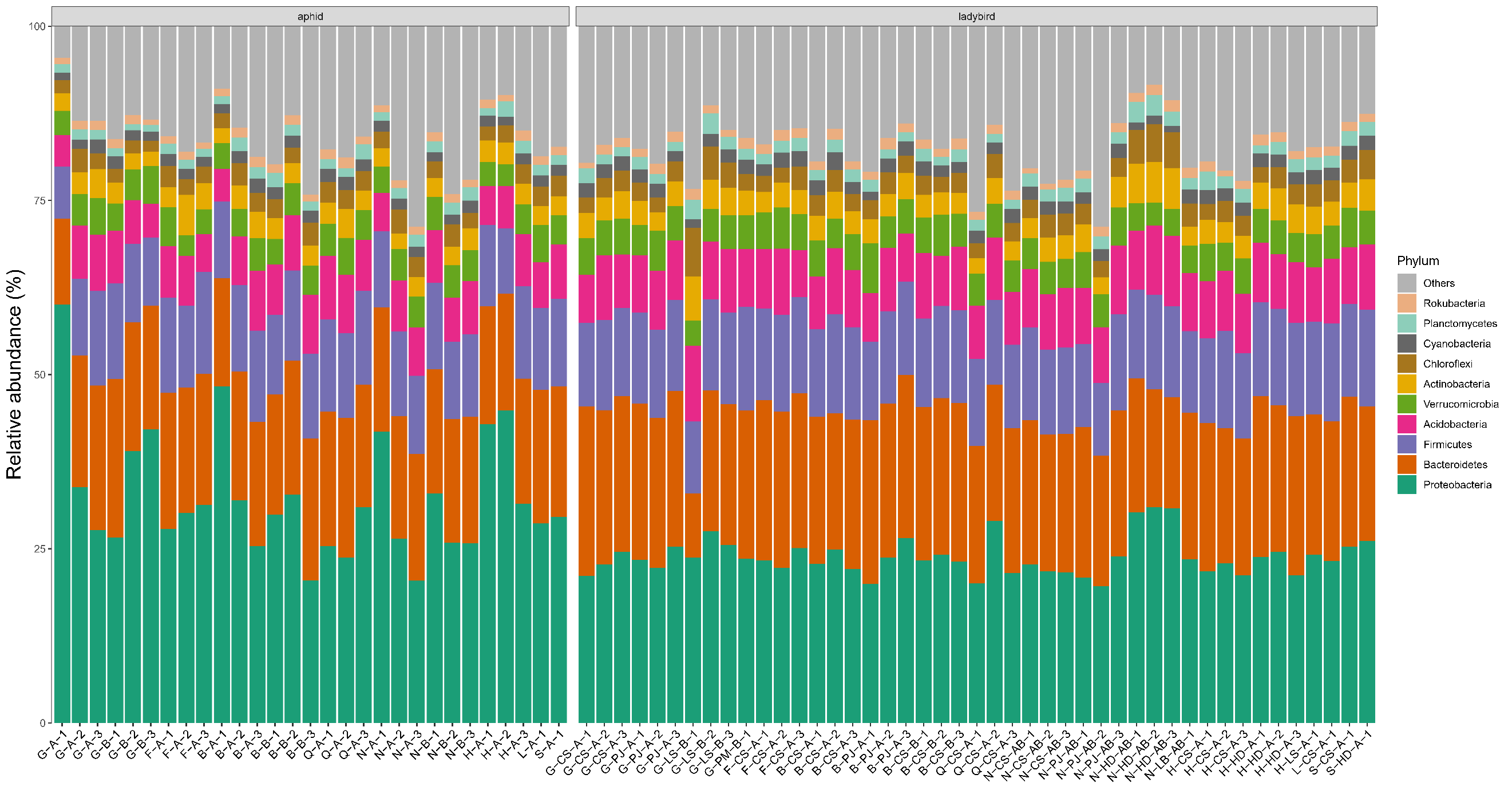


Figure S2 Phylum-level taxonomic of aphid and ladybird samples collected from various locations in Guangxi, China. Detailed information on sample codes is provided in Table 1.


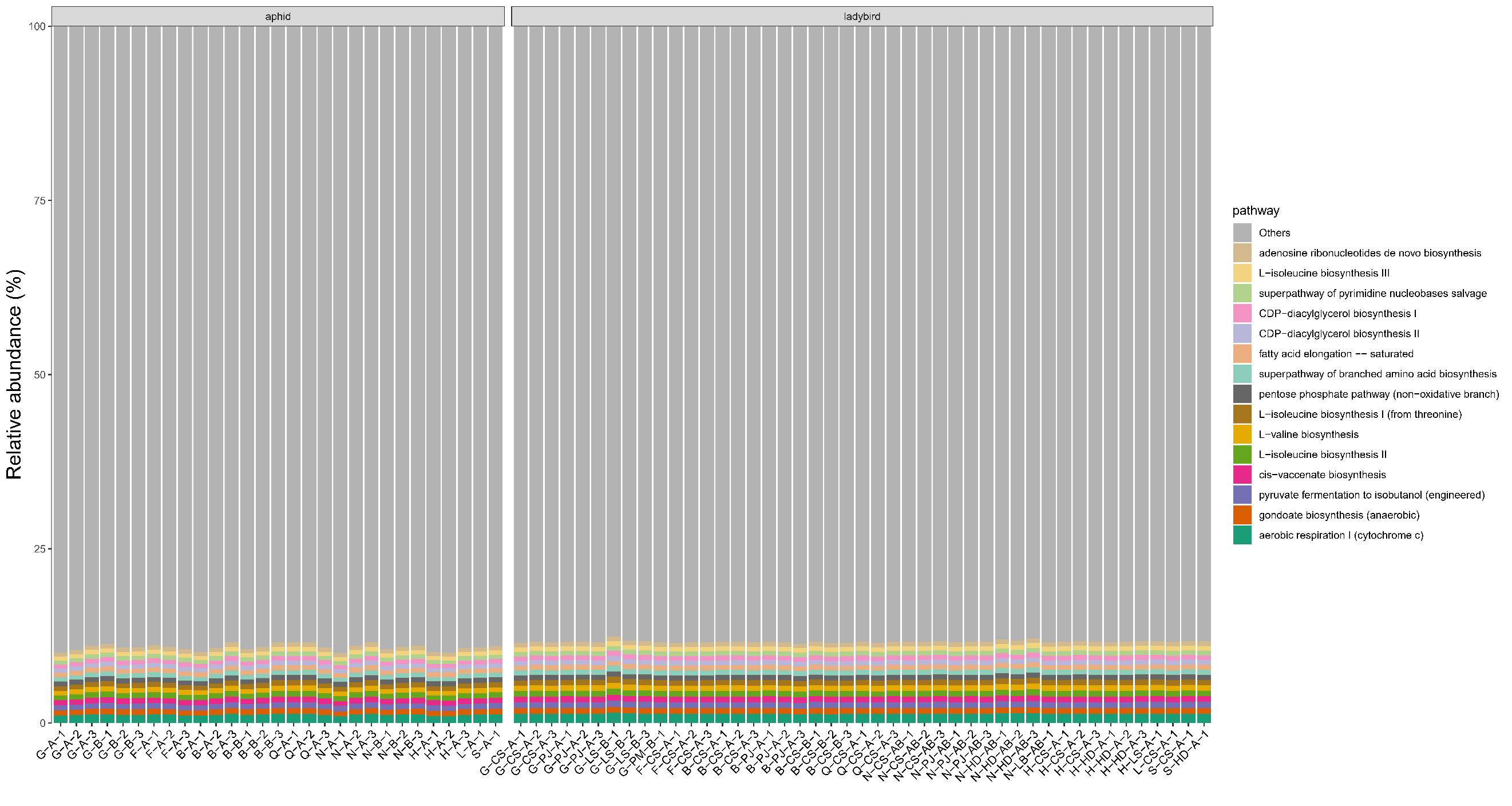


Figure S3 Functional abundance of the top 15 pathways in the microbiomes of aphid and ladybird samples collected from various locations in Guangxi, China. Detailed information on sample codes is provided in Table 1.


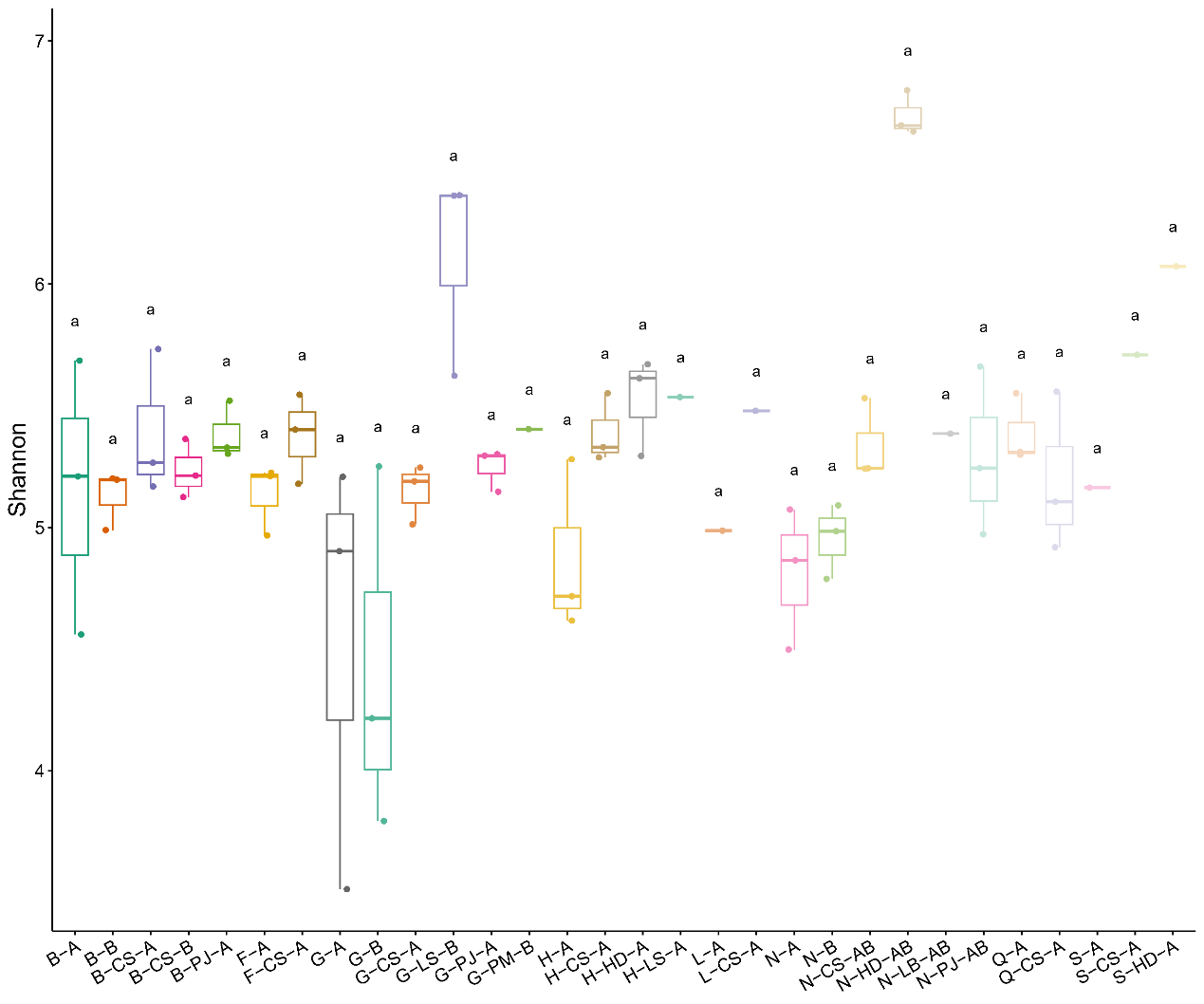


Figure S4 Scattered boxplots of the Shannon index for different populations of aphid and ladybird samples. Detailed information on sample codes is provided in Table 1.


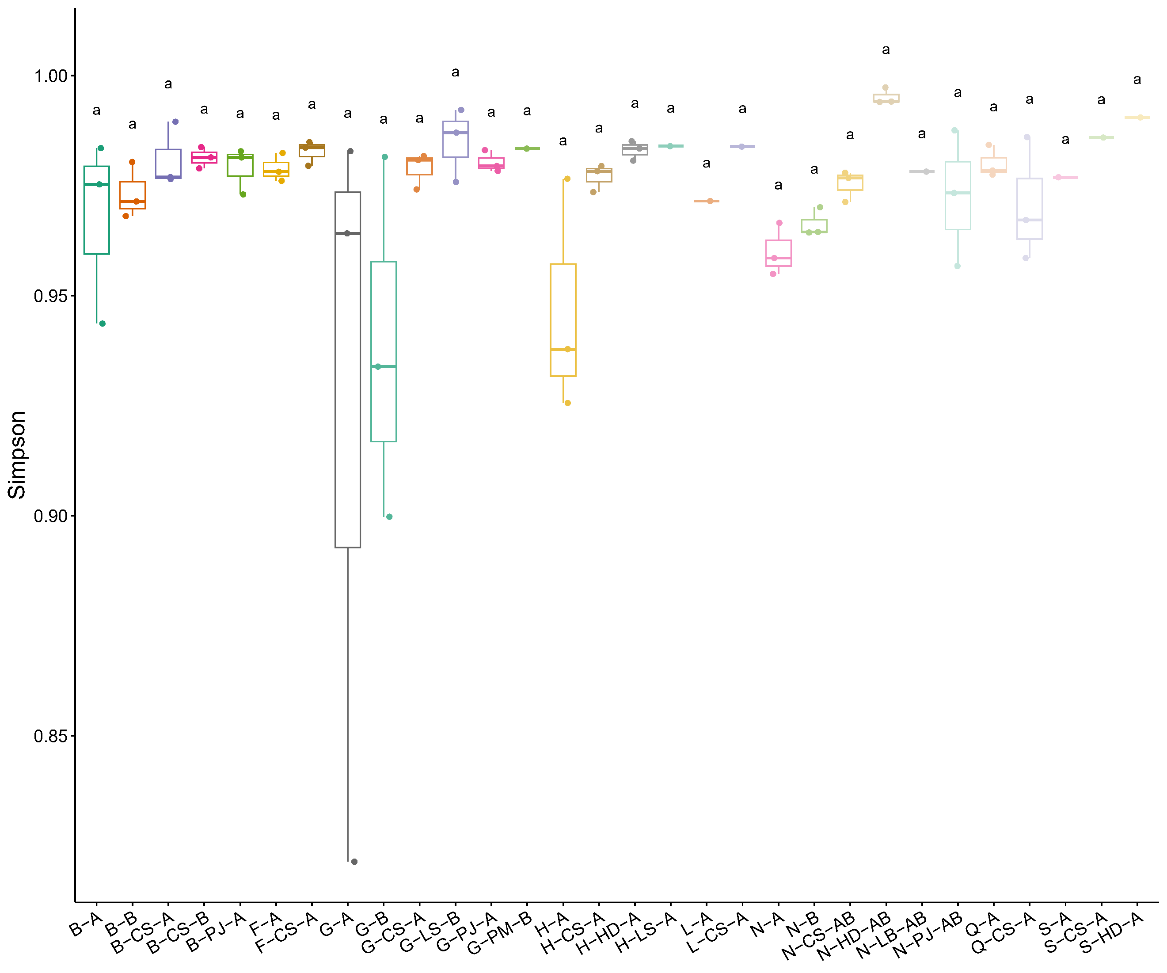


Figure S5 Scattered boxplots of the Simpson index for different populations of aphid and ladybird samples. Detailed information on sample codes is provided in Table 1.


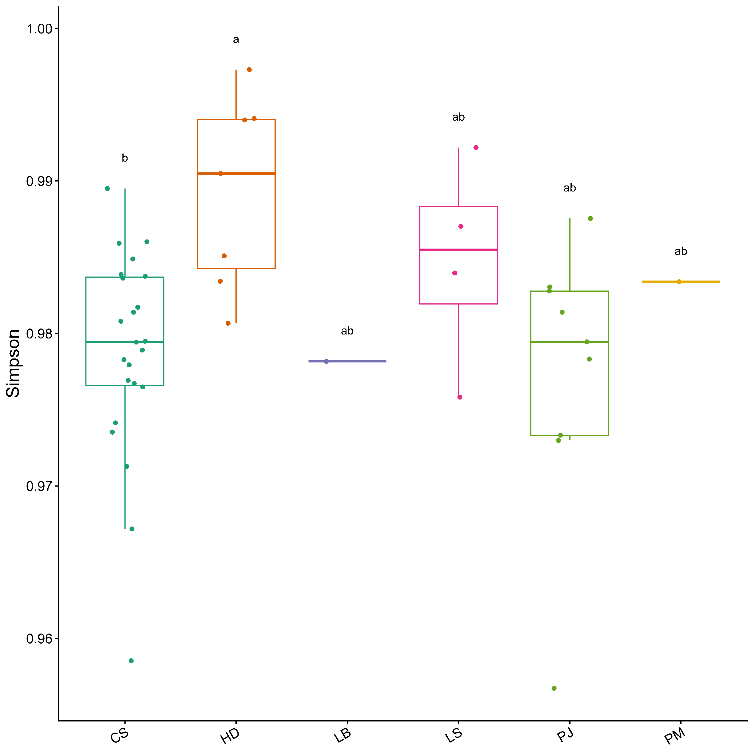


Figure S6 Scattered boxplots of the Simpson index for different ladybird species. CS: *Cheilomenes sexmaculata*, HD: *Harmonia dimidiata*, LB: *Lemnia biplagiata*, LS: *Lemnia saucia*, PJ: *Propylea japonica*, PM: *Platynaspis maculosa*.


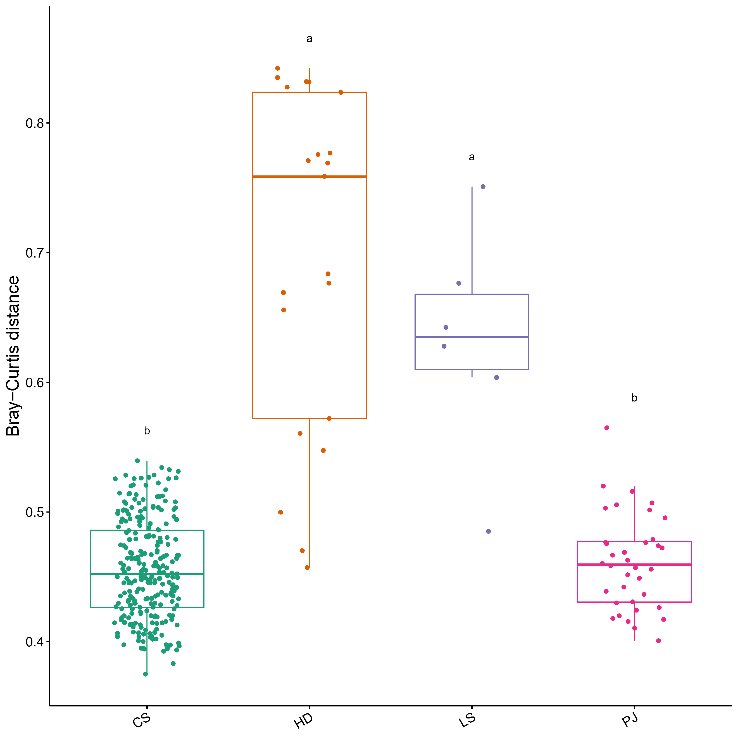


Figure S7 Scattered boxplots of Bray-Curtis distances within the same ladybird species. CS: *Cheilomenes sexmaculata*, HD: *Harmonia dimidiata*, LS: *Lemnia saucia*, PJ: *Propylea japonica*.


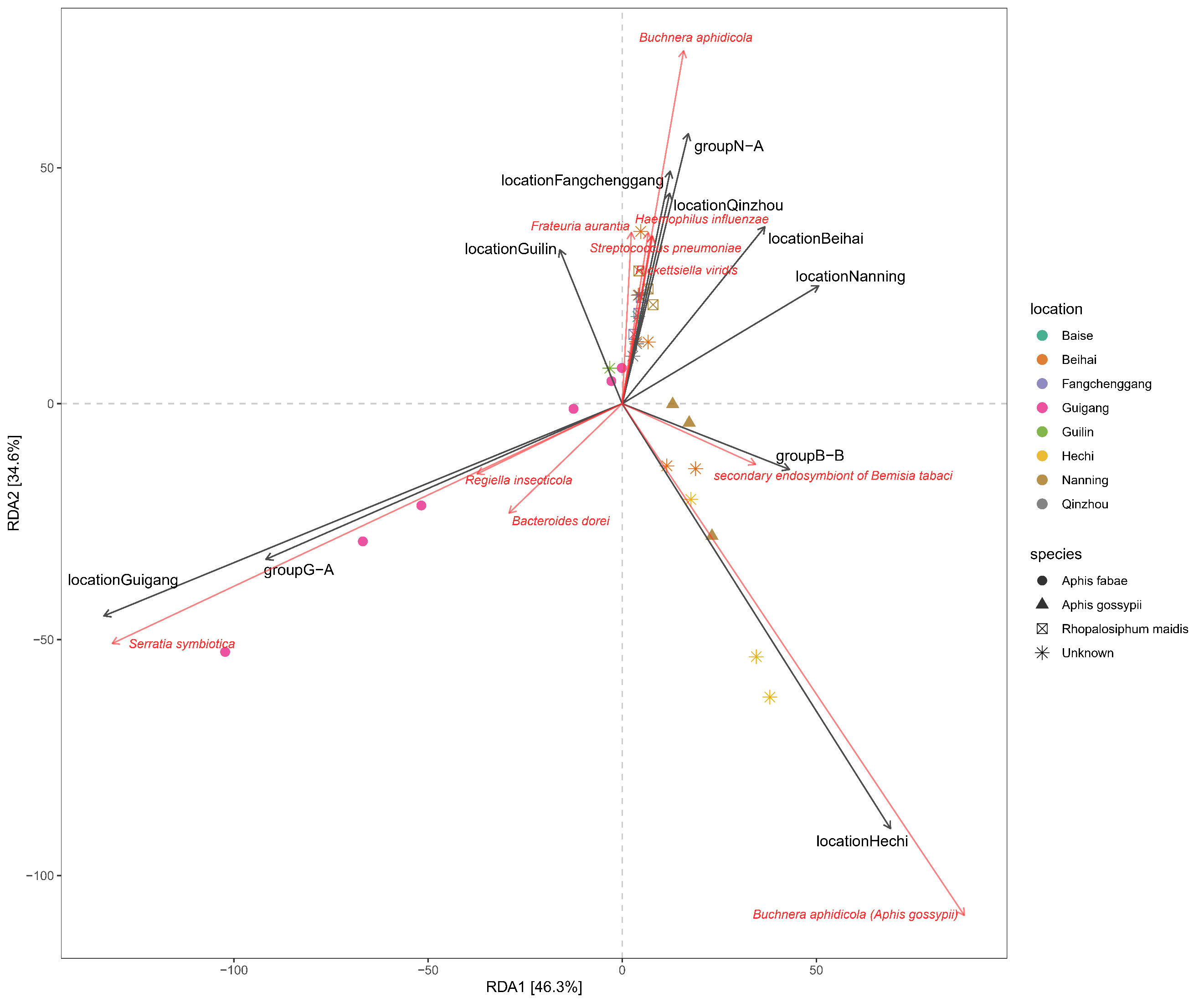


Figure S8 Redundancy analysis (RDA) of microbial composition for aphid samples, constrained by location and population. Detailed information on population (group) codes is provided in Table 1.


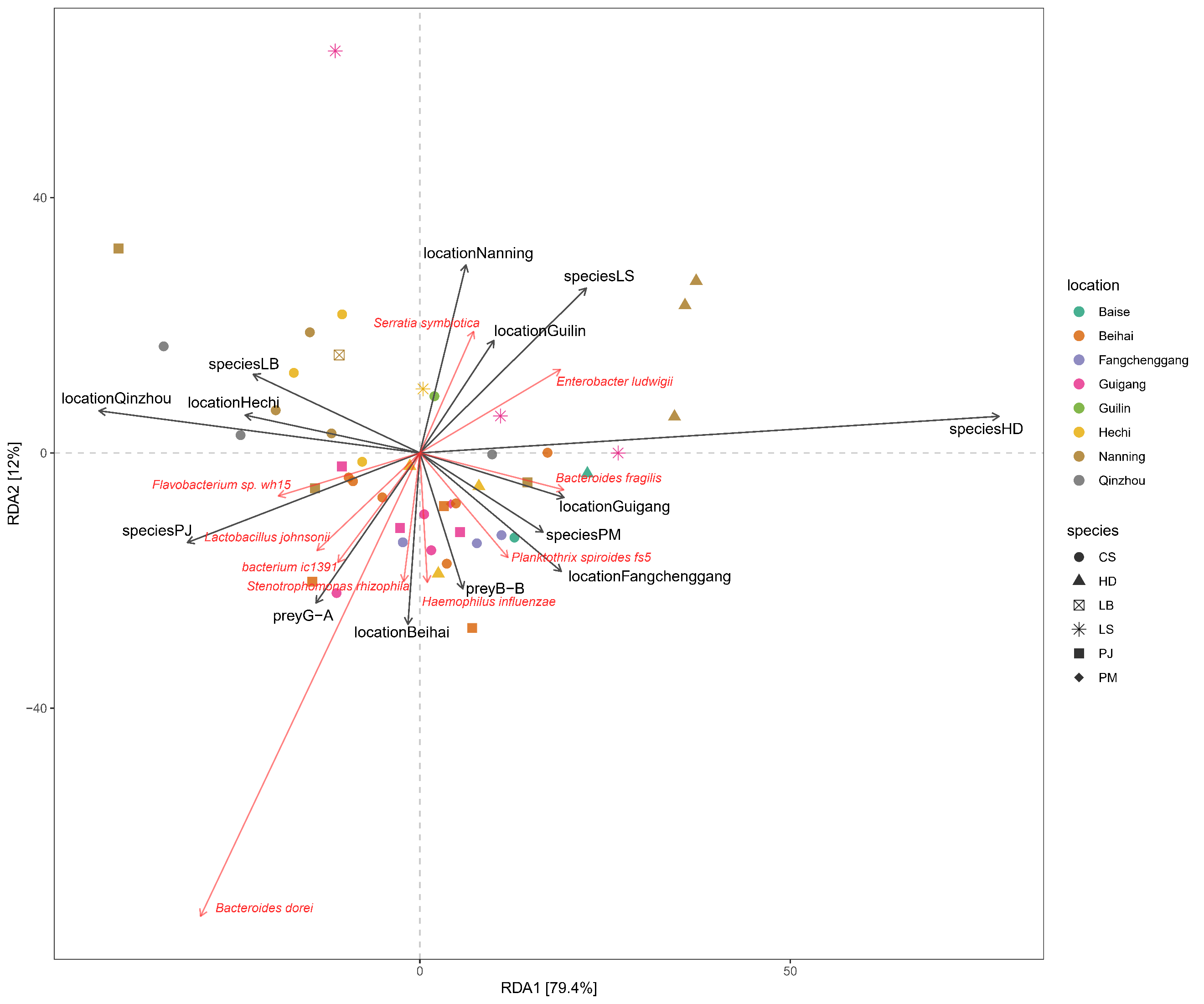


Figure S9 Redundancy analysis (RDA) of microbial composition for ladybird samples, constrained by species, location and prey population. Detailed information on prey population codes is provided in Table 1.


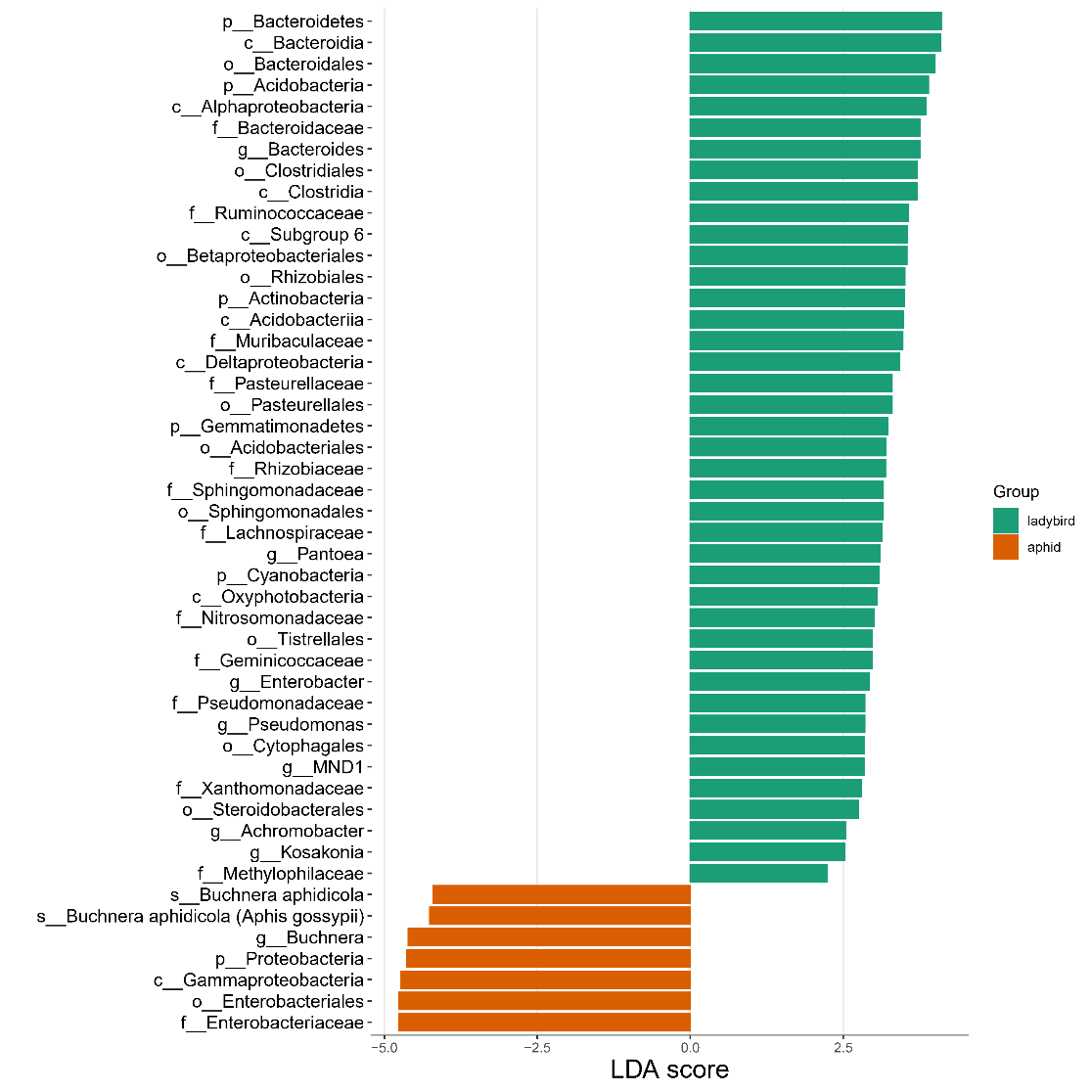


Figure S10 Taxa with significantly higher abundance in ladybird or aphid samples identified using the linear discriminant analysis (LDA) effect size (LEfSe) method.
